# Meanings attributed to pregnancy by women with Systemic Lupus Erythematosus: a grounded theory

**DOI:** 10.1590/1980-220X-REEUSP-2024-0413en

**Published:** 2025-07-07

**Authors:** Rebeca Rosa de Souza, Mayckel da Silva Barreto, Elen Ferraz Teston, Monika Wernet, Maria Eduarda Pascoaloto da Silva, Alexandrina Maria Ramos Cardoso, Sonia Silva Marcon

**Affiliations:** 1 Universidade Estadual do Paraná, Departamento de Enfermagem, Paranavaí, PR, Brazil.; 2 Universidade Federal do Mato Grosso do Sul, Departamento de Enfermagem, Campo Grande, MS, Brazil.; 3 Universidade Federal de São Carlos, São Carlos, SP, Brazil.; 4 Escola Superior de Enfermagem do Porto, Porto, Portugal.

**Keywords:** Lupus Erythematosus, Systemic, Pregnancy, High-Risk, Autoimmune Diseases, Nursing, Grounded Theory, Symbolic Interactionism

## Abstract

**Objective:**

To construct a substantive theory about the meanings attributed to the experience of pregnancy by women with Systemic Lupus Erythematosus.

**Methods:**

Qualitative study using Symbolic Interactionism as a theoretical framework, and Grounded Theory, a constructivist approach, as a methodological framework. Data collection took place between January and August 2022 through in-depth interviews, which were video and audio-recorded and carried out remotely. The theoretical sample consisted of 27 participants, distributed into four sample groups.

**Results:**

From constant comparative analysis the central phenomenon was constructed: *Understanding reproductive planning as the key to the gestational process in Systemic Lupus Erythematosus*, consisting of two categories, which show divergent experiences and meanings based on whether or not the pregnancy was planned.

**Conclusion:**

Personal interaction with the phenomenon, based or not on the “pillars” of reproductive planning, and social interaction with the multidisciplinary health team reflect on the way the woman perceives, behaves, and gives meaning to pregnancy. Thus, it is reiterated that reproductive planning is considered the key to gestational outcome in SLE.

## INTRODUCTION

Considered an important risk factor during the pregnancy- puerperal process, Systemic Lupus Erythematosus (SLE) stratifies pregnancy as high risk. Therefore, women with this disease have to plan their pregnancy and be monitored by a multidisciplinary health team^(1,2,3)^, considering that medication adjustment, as well as prenatal care, must be started immediately^(4,5)^.

The manifestations of SLE during pregnancy and pregnancy during disease activity are topics widely discussed in the literature^(6,7,8,9,10)^, whose studies showed an increase in disease activity during pregnancy and in the postpartum period^(8,9,10,11)^. However, the risks of exacerbations appear to be greater when SLE has been active for at least six months prior to preconception. Likewise, risks are reduced when conception occurs with the disease in remission^(11)^.

Pregnancy, being high risk, is perceived by women with SLE as a complex process that triggers feelings of fear, anguish and uncertainty^(2,3,8,10)^. Thus, this phenomenon is signified based on experiences such as the presence of complications due to the activation of the disease, repeated miscarriages, premature births, pre-eclampsia, fetal malformation, and complications of Antiphospholipid Syndrome (APS), which certainly impact the way women live this experience^(12)^. However, the health care received also constitutes part of this process^(1,13)^.

In view of this, the need to understand in depth the meanings attributed to pregnancy by women with SLE is highlighted, considering their personal interaction with the phenomenon, as well as their social interaction with the multidisciplinary health team. Thus, the objective was defined as constructing a substantive theory about the meanings attributed to the experience of pregnancy by women with Systemic Lupus Erythematosus.

## METHOD

### Study Design/Theoretical and Methodological Framework

Explanatory research, of a qualitative nature, which had as its theoretical-conceptual basis Symbolic Interactionism (SI), which assumes that meanings are attributed based on lived experience^(14,15)^. As a methodological framework, the Grounded Theory (GT) with a constructivist perspective was adopted, which considers that the experiences of the researcher and participant in the context are relevant for the interpretation of the phenomenon^(16)^. In describing the study results, the Consolidated Criteria for Reporting Qualitative Research (COREQ) guidelines were used^(17)^.

### Local

The women with SLE participating in the study were found in the private group “Lúpus Brasil – o desabafo” (Lupus Brazil – the confession), hosted on the social media Facebook®, created in 2014, which has more than 30 thousand members. The group’s objective is to encourage the exchange of information and experiences, with pregnancy being a recurring topic of discussion.

### Population

Twenty-seven women participated, who were divided into four sample groups (SG). The members were invited through a public post in the group “Lúpus Brasil – o desabafo” and text messages sent to those who participated in the publications and made comments about SLE and pregnancy.

### Selection Criteria

The inclusion criteria were: being 18 years of age or older, having experienced pregnancy with SLE; and having access to the internet with a technological device for video call. The only exclusion criterion established was presenting some difficulty in communication, with one woman being excluded due to deafness.

### Sample Definition

To form the first SG, the following hypothesis was considered: the meaning attributed to pregnancy is related to the way the woman experiences the phenomenon. This SG was intentionally formed by 14 women with SLE who experienced high-risk pregnancies. During data collection and analysis, it became clear that the gestational experience differs and/or changes when the woman experiences other associated clinical conditions. Among these, APS was frequently reported, and this seemed to mark a change in behavior and outline the perception about pregnancy.

Based on this data, the following hypothesis was formulated: the meaning attributed may differ as the woman experiences another clinical condition with potential risk of gestational complications. Therefore, the second SG consisted of six women who had thrombophilia during pregnancy. Data analysis of this group showed that the risk of pregnancy loss or the experience of abortion seemed to influence the meanings of the experience, raising the following hypothesis: the way in which a woman understands pregnancy can be modified when she experiences pregnancy losses.

Thus, the third SG consisted of four women with SLE who suffered spontaneous abortions. Among the participants in this group there was a woman who experienced pregnancy in Italy, and thus the hypothesis arose: becoming pregnant with SLE outside the country of origin can change the way a woman understands pregnancy. Accordingly, the fourth SG was made up of three Brazilian women who experienced pregnancy as immigrants in other countries.

The constitution of the sample groups was finalized based on the theoretical saturation of the data, that is, the new interviews and the new data collected did not bring new insights into the central phenomenon constructed, thus understanding that the objective of the investigation was achieved, finalizing the recruitment of new participants.

### Data Collection Period

Data were collected between January and August 2022, through in-depth interviews conducted via video call. These were video and audio recorded after authorization and had an average duration of seventy-five minutes. The applications *WhatsApp and Messenger* and the video communication platform *Google Meet* were used.

In all interviews, a guiding question appropriate to each SG was used, as follows: tell me about your pregnancy with SLE; tell me about being pregnant with SLE and thrombophilia; tell me about your miscarriages in pregnancies with SLE; tell me about being pregnant with SLE outside your country of origin. All interviews were conducted by a nurse, a PhD student in Nursing with experience in qualitative research and who experienced repeated spontaneous abortions and a pregnancy with thrombophilia.

### Data Analysis

**Figure 1:**
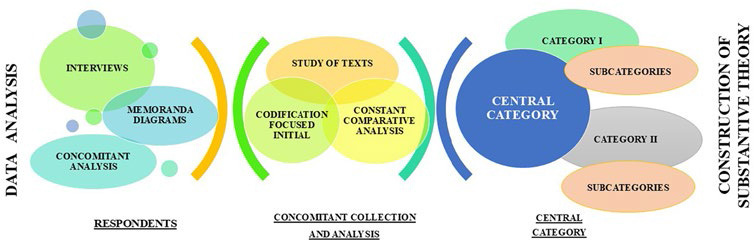
Diagram of the data analysis process, production of the central category, categories and subcategories – Maringa, PR, Brazil, 2023.

Data were organized and analyzed using MAXQDA software *plus* 2020. The interviews were transcribed and submitted to text study, accompanied by the development of memos and diagrams. Initial coding occurred word by word, line by line and incident by incident, using the constant comparative method, generating initial and provisional codes. Focused coding was carried out, using the most significant and frequent initial codes, comparing the initial codes of the same interview with those of subsequent interviews, aiming to create more targeted, selective, and conceptual codes. This allowed the separation, classification, integration and organization of data, aiming at the conceptualization of the empirical material into subcategories and, subsequently, the identification of the categories conceptual properties. Figure 1 presents the data analysis process, production of the central phenomenon, categories and subcategories that made up the substantive theory.

### Ethical Aspects

The development of the study was approved by the Permanent Human Research Ethics Committee of the signatory institution, opinion no. 5.132.482, CAAE: 53038321.8.0000.0104, following Resolution no. 466/12 and the guidelines for research in a virtual environment of the National Commission for Ethics in Research. The Free and Informed Consent Form (FICF) was signed by *Google Forms.* The speech excerpts were identified by the letter G referring to the sample group and the respective number, followed by the letter P (participant) and the number corresponding to the interview order. Example: (G1, P3).

The criteria of adjustment, understanding, generalization and control were evaluated positively, and it was possible to see that the diagrammatic representation of the theory was satisfactory. *I can see myself in both situations where, during an unplanned pregnancy, I ended up having a lot of complications. When I had a planned pregnancy and was accompanied by professionals, I had a more peaceful pregnancy and managed to reach the end.* (P1). *Pregnancy planning was essential for my son’s birth, it avoided losses and suffering, I can see that in theory* (P4). *Analyzing the theory, it is possible to see that the most important thing is pregnancy planning. It was like that with me, I lost the first one, then I managed to have my son* (P6).

In terms of control, the evaluators reported that the theory clearly revealed the relationship between Lupus and pregnancy. *For those who have experienced both, the theory makes sense and presents what happens during a pregnancy with Lupus.* (P1)*. It is the truth and necessity shown in a figure, it shows the importance of a planned pregnancy* (P2).

After this process, the theory was refined in terms of aesthetic improvement and technical terms, seeking more consistency in its presentation.

### Theory Validation

First, an internal validation was carried out, in which the theory was compared with the data interpreted in the initial stage, aiming at improving the interpretations and identifying whether the theory presented was explanatory of the findings. Later, the theory was presented, remotely by *Google Forms,* to a group of six evaluators, all of whom were participants in the study. The evaluation followed the structure recommended by Strauss: Adjustment, Understanding, Generalization, and Control. Validation took place on February 2023.

## RESULTS

Twenty-seven women aged between 24 and 46 years old, living in four Brazilian regions, in 11 states and three in other countries (Germany, Japan and Sweden) participated. The time since diagnosis of the disease varied from two to 28 years, and the number of pregnancies from one to six, with five being the maximum number of miscarriages. The main comorbidities secondary to SLE experienced were: APS (23), Rheumatoid arthritis (3), Fibromyalgia (3), Sickle cell anemia (1), Hypothyroidism (1), Reynaud’s syndrome (1), Diabetes Mellitus (1), and Lupus nephritis (1).

**Figure 2:**
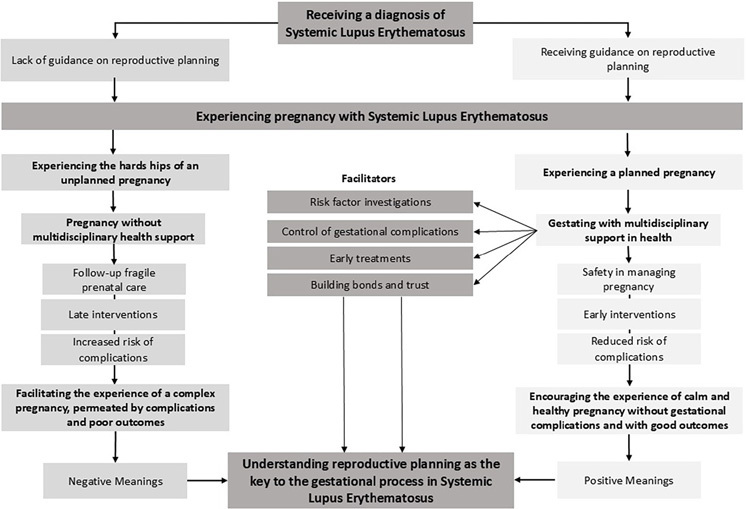
Substantive theory presentation diagram: Understanding reproductive planning as the key to the gestational process in Systemic Lupus Erythematosus – Maringá, PR, Brazil (authors 2023).

Figure 2 presents the central phenomenon, *Understanding reproductive planning as the key to the gestational process in Systemic Lupus Erythematosus,* consists of two related categories, which portray the experience of planned and unplanned pregnancies.

### Understanding Reproductive Planning as the Key to the Gestational Process in Systemic Lupus Erythematosus

The meaning attributed to pregnancy in the context of SLE is established based on the way the phenomenon presents itself in the woman’s life, which, in turn, is mediated by the meanings already attributed to the disease and which changes with the lived experience, and based on social interaction with the world around her. Thus, the meaning attributed to pregnancy by women with the same chronic condition can be completely different, depending on whether or not it was planned.

### Experiencing a planned pregnancy

Planned pregnancy is possible when the woman has adequate knowledge about Lupus and its implications. Receiving guidance can have a positive impact on the construction of the “social self” and influence behaviors, experiences and the meanings attributed. *Receiving guidance about pregnancy was critical, because I had the opportunity to prepare myself for that moment. I learned about my illness and what I needed to do to have a good pregnancy* (*G1, P2*)*. Since I was a little girl, my rheumatologist told me that I would have to take care of myself and that I couldn’t get pregnant without planning it first, I grew up with that in my mind. Pregnancy was not easy, but I already knew the risks and what could happen and that helped me* (G2, P20).

By experiencing the phenomenon in a planned way, with the support of health professionals, pregnancy is experienced with greater safety and tranquility, as it reduces the risks of complications and unfavorable gestational outcomes. *Throughout the pregnancy, my rheumatologist and gynecologist were with me, they took care of me, we even became friends because we talked so much and that helped a lot, it was essential* (G2, P15). *During my second pregnancy, I had professional monitoring, a rheumatologist, a high-risk obstetrician. Because of this, I had a more peaceful pregnancy, with fewer complications.* (G1, P14) *After having gone through both experiences: an unplanned pregnancy and a planned one, I can say that I feel like a winner, today I understand how important it is to plan a pregnancy when you have Lupus* (G1, P7)*. Being pregnant with Lupus means being a warrior to endure and make it to the end. Because it’s one day at a time, we don’t know if the child will still be there the next day, so we have to have faith and a lot of strength. Feeling supported by your medical team, your family and following the treatment and care correctly are very important. Today I believe that pregnancy is possible, it is not easy, but possible. It is necessary to plan* (G1, P12).

Reproductive planning provides an opportunity to achieve the dream of motherhood and the shared decision between the woman and the health professional about the appropriate time to become pregnant is essential. *Before getting pregnant again, I planned the pregnancy, together with a rheumatologist and gynecologist, this was essential. We planned it and I only got pregnant when my disease was in remission.* (G2, P17). *My pregnancy was completely planned, I had been preparing for it for a long time. When I decided, I looked for the medical team and I was very well assisted. This was one of the most important points* (G4, P 25). *When I got pregnant, my Lupus had been in remission for over a year, and that’s a big deal. If you want to have children and you have Lupus, you have to wait for the disease to go into remission. My doctor helped me decide the right time to get pregnant* (G2, P16).

This is because it allows for psychological preparation, especially when there is a condition requiring complex treatments. *Treating thrombophilia is not easy, nobody likes injections. But I accepted it well. I already knew that when I got pregnant I would have to treat it and this helped me. We feel victorious, it’s a huge achievement to go through this*. (G2, P19). *Those injections symbolize the love, strength and courage of a mother in pursuit of her dream. It’s an act of love to pierce yourself every day. Seeing your body bleed, the huge bruises that form. We place all our hope in that injection* (G2 P15).

The assistance provided by different professionals was also valued by Brazilian women living in other countries, demonstrating that feeling supported is essential. *I was very confident, I didn’t feel afraid, I received a lot of information. I had a lot of support from my medical team. I knew I was well looked after, and that made a difference.* (G4, P25)*. I had my prenatal care with a multidisciplinary team, I saw her virtually every week, I did tests, lots of check-ups, and that’s how it was until the end of pregnancy. I created a good bond with the team, and that was essential.* (G4, P27).

Such experiences, in addition to contributing to the construction of positive meanings, are responsible for providing a new perception about health professionals. *Since I was a little girl, I heard that I would never be able to get pregnant. I spent my entire adolescence hearing negative things from doctors. Today, we realize that they have improved. I haven’t heard it that often anymore, that one bad thing, and that’s very important. It makes us feel better. It’s great to know that today’s professionals don’t say the same things as those of the past* (G4, P27).

### Experiencing the Hardships of an Unplanned Pregnancy

When experiencing an unplanned pregnancy full of complications, women attribute negative meanings to the experience. *For me, being pregnant with Lupus is horrible. I felt very bad, very depressed, I felt like giving up on everything. It was horrible* (G1, P9). *I do not recommend pregnancy to a woman with Lupus, never in my life, it was the worst phase of my life. I thought I was going to die. I don’t want to have children any more* (G4, P26).

Some women with SLE did not believe they could get pregnant and for this reason did not worry about making reproductive plans. *I spent my life believing that I would never have children. That a woman with Lupus cannot have children. When I found out I was pregnant, I was shocked, afraid of what could happen to me and the baby. It was maddening* (G2, P19).

This conception is the result of erroneous guidance received throughout life, including from health professionals. *I grew up hearing that women with Lupus don’t have children, and that stayed with me for a long time. When I met my husband, I even said to him: look, I have Lupus and I will never be able to give you a child. I really believed that. Because that’s what doctors taught me my whole life* (G4, P27). *I felt very bad, feelings of incapacity, of failure. It’s every woman’s dream to be a mother. It’s very difficult to spend your life hearing that you can’t have children.* (G3, P22).

The lack of adequate professional guidance, particularly regarding reproductive planning, is a source of feelings of worthlessness and misinformation. *I felt like the worst woman in the world. I tried so many times and every time I failed I heard: you are going to die, it is easier to die than to be able to have a child with Lupus. This hurt me a lot. Because it was my dream to be a mother, I felt very sad, incapable* (G3, P23). *I had never received any guidance about pregnancy, what I knew was what I looked up on the internet. No doctor advised me on this. So, when I got pregnant, I had a lot of complications, I went to the ICU, I almost died and I would have died if I hadn’t been lucky enough to be treated in a good hospital* (G1, P10). *I got pregnant without planning. I didn’t know much about my illness, I didn’t even know that I had to plan, and then all this happened, I lost my baby, I got very sick, I suffered a lot* (G3, P21).

From the perspective of not being able to get pregnant, many women do not seek reproductive planning and end up suffering gestational complications that negatively impact their lives and the meaning attributed to them. *I had six pregnancies and five miscarriages, one after the other. It was very difficult to go through all these losses. As I did not treat it properly in the other pregnancies, I lost it, and only in the last one with all the correct treatments did it work out* (G3, P 23). *I got pregnant the first time with an active disease, I felt very ill, the Lupus got worse, I stayed in hospital most of the time, I had a stroke, hypertension, pre-eclampsia, and I lost my baby. It was a very difficult process* (G1, P12). *My two children were born prematurely at 32 and 34 weeks, they were hospitalized and I almost died.* (G1, P11).

Underlying diseases such as APS are experienced, which require complex treatments. *Treating APS is very difficult. Injection every day. We get all bruised, pain, it’s very painful.* (G2 P18)*. I don’t even like to remember it, it was the worst part of it all. Those injections hurt, they bleed. I don’t know if I would have the courage to face them again.* (G4 P25). *I’ve always been terrified of needles, that was definitely the hardest part. I never had the courage to apply it to myself, it was always my husband* (G2, 17).

Weaknesses in care were also noticed by women who experienced pregnancy in other countries. *Here in Sweden, doctors are also against pregnancy in women with Lupus, they are horrified when a woman with Lupus becomes pregnant. We realize that there is a lack of knowledge. They don’t have the security to accompany such a pregnancy* (G4, P 25)*. Here in Japan, abortion is allowed, if you have Lupus you can have an abortion. They are completely against pregnancy in women with Lupus and are not prepared to provide care. My experience of having a child here was horrible, terrifying.* (G4, P 26).

The meanings attributed to unplanned pregnancy are negative and have a direct relationship with the process experienced. *It was a turbulent pregnancy, lots of pain, lots of hospitalizations, until the birth in the seventh month.* (G1, P 11). *The experience I had was horrible, unfortunately. I don’t want to get pregnant ever again in my life* (G4, P26).

## DISCUSSION

The meanings attributed to pregnancy are influenced by reproductive planning or its absence. Furthermore, they are related to the way women experienced the gestational process, with personal interaction and social interaction being the structuring bases for the construction of these meanings. In this sense, the results corroborate the perspectives of SI, which emphasize that meaning is created from the way in which subjects process experiences, how the phenomenon presents itself in their lives and how they and their reflections are interpreted^(14,15)^.

It was possible to understand that when experiencing a planned pregnancy with the support of a multidisciplinary health team, the meanings attributed by women based on the interaction of the self, the mind and society are positive, in the sense that they were able to overcome the challenges and limitations of SLE and associated conditions. This is because these women experienced a calmer and healthier pregnancy, and at the same time, felt supported and safe.

A retrospective cohort study conducted in Campinas, São Paulo, Brazil, aimed at evaluating the effects of pregnancy in patients with SLE, showed that when women carry out reproductive planning and become pregnant with the disease in remission, the gestational process is experienced more smoothly, with less risk of complications and greater chances of positive outcomes^(12)^.

Scientific literature emphasizes the importance of reproductive planning^(12,18,19)^ and the positive implications in relation to this process, such as: reduction of complications; early diagnosis and treatment of risk factors and underlying diseases; and better physical and psychological preparation, as well as the possibility of planning childbirth and the postpartum period^(12,18,19)^.

When there is a bond and trust between the pregnant woman and the professionals who monitor the high-risk pregnancy, the obstetric outcomes, as well as the management of the gestational process, are experienced in a more positive way^(9,13)^. This relationship predisposes to decision-making that favors specialized and humanized care, whose objective is the safety and quality of care, as well as the empowerment of women during the gestational period.

The interaction between the woman and the professional who monitors her health condition allows pregnancy to occur at an opportune time, reducing risk factors. This is because the success of pregnancy, in most cases, is linked to the beginning of pregnancy at an opportune time with the disease in remission for at least six months before conception^(10,13,18,19)^.

Thus, when planned, the phenomenon is experienced in a positive way, because, in addition to being better prepared personally, the woman will be able to count on the support of a multidisciplinary health team, which contributes to a healthier experience and, consequently, its re-signification. According to SI, this social interaction, which occurs over time, allows women to assume their identity, organize their behavior, position themselves in relation to the clinical condition and symbolize their experiences. Thus, social interaction is understood as one of the main tools to support the outcome of pregnancy and also to create meanings about the process experienced^(14,15)^.

However, most of the time, pregnancy in women with SLE occurs without reproductive planning, which can trigger complex experiences, permeated by complications^(1,2,3)^. This happens because they spent part of their lives believing that they could not get pregnant or because they did not receive adequate guidance from professionals. In this way, concepts, behaviors and attitudes are established based on these teachings, resulting in unplanned pregnancies that are not monitored by specialized health professionals.

There was a period when medical advice was that women with SLE should avoid pregnancy, as this represented a high risk of maternal and fetal complications. However, with advances in science, professional training, greater diagnostic accuracy and therapeutic resources, pregnancy in women with SLE is now completely viable^(12,18,19)^. However, they are considered high-risk pregnancies and require, in addition to special monitoring, the existence of a bond and trust between the patient and health professionals^(12,18)^.

Some women in the study, however, reported weaknesses in social interaction with professionals, giving rise to the idea that the pregnancy occurred without planning due to a lack of guidance and specific knowledge. Other studies have already referred to the precariousness of the assistance provided to women with SLE, highlighting the lack of guidance on reproductive planning and professional pessimism regarding the combination of SLE and pregnancy^(1,13)^. Sometimes, this precariousness in care is a result of the professional focus on drug treatment of the disease, making the desires, dreams, and ideals of these women invisible.

These care characteristics have a negative impact on the occurrence of unplanned pregnancies and traumatic experiences^(1,2,3)^. Furthermore, in the presence of APS, treatment becomes even more complex, requiring daily applications of injectable anticoagulants, which trigger pain, bruising and bleeding^(20)^, contributing to a negative perception of the experience.

Thus, the construction of the “Self” from this experience is, for the most part, negative, since the process is experienced in a peculiar way, permeated by feelings of fear, anguish, and uncertainty. The lack of social interaction or impaired social interaction with the multidisciplinary team produces even more negative impacts. Therefore, it becomes important to provide visibility to women and their desires throughout the follow-up, so that one can understand that desires/dreams can change depending on the stage of the disease and the risk factors involved.

It is important to highlight that the monitoring of a woman with SLE should be carried out from the preconception period until the puerperium^(5,21)^, with the aim of early investigation of risk factors and diseases that may impact gestational outcomes^(5,13,22)^. Preconception counseling has to include guidelines such as: risk of disease exacerbation and potential obstetric, fetal, and perinatal complications associated with unplanned pregnancy; risks of prematurity; investigation of early clinical situations; the importance of pregnancy with the disease in remission, as well as the importance of high-risk prenatal monitoring^(5)^.

With the advancement of science and scientific knowledge about this phenomenon, reproductive planning is increasingly gaining prominence and indication, and should be identified as one of the ways to reduce morbidity and mortality and increase the success of these pregnancies^(9)^. Thus, planning pregnancy with SLE and reproductive surveillance of women increase the chances of good gestational outcomes, contributing to the construction of positive meanings.

A possible limitation of the study is related to selection bias, since women with SLE were located on a virtual platform, which limited the participation of those who did not have access to the internet. However, the results of this study contribute to scientific knowledge about pregnancy in women with SLE and provide support for discussions about healthcare aimed at this population, as well as for understanding the importance of reproductive planning for pregnancies with this chronic condition. It is suggested that future research investigate the role of the family in this phenomenon.

## CONCLUSION

The construction of this substantive theory concluded that the meanings attributed to pregnancy by women with SLE differ based on their lived experience, which in turn is influenced by whether or not the pregnancy was planned. The experience of a planned pregnancy with multidisciplinary health support is seen as positive, as it provides favorable gestational outcomes. Conversely, unplanned pregnancy experienced without multidisciplinary support or with fragile support is negatively signified, especially due to the experience of complications, the construction of negative feelings and unfavorable gestational outcomes.

Personal interaction with the phenomenon is directly affected by whether or not the woman with SLE planned her pregnancy and the social interaction established with the multidisciplinary health team in monitoring her in the health/disease/pregnancy process. These directly influence the way a woman perceives, behaves and understands pregnancy. Thus, reproductive planning is perceived as the key to gestational outcome in SLE.

## Data Availability

The complete set of data supporting the results of this study was published in the article itself.
