# Supplementary material for: Meanings attributed to pregnancy by women with Systemic Lupus Erythematosus: a grounded theory
Source: Rev Esc Enferm USP. 2025 Jul 7;59:e20240413. doi: 10.1590/1980-220X-REEUSP-2024-0413en (PMC12245392; doi:10.1590/1980-220X-REEUSP-2024-0413en)
Supplement: Translation

# Translation: Significados atribuídos à gestação por mulheres com Lúpus Eritematoso Sistêmico: uma teoria fundamentada nos dados

**DOI:** 10.1590/1980-220X-REEUSP-2024-0413pt

**Authors:** Rebeca Rosa de Souza, Mayckel da Silva Barreto, Elen Ferraz Teston, Monika Wernet, Maria Eduarda Pascoaloto da Silva, Alexandrina Maria Ramos Cardoso, Sonia Silva Marcon

**Affiliations:** 1 Universidade Estadual do Paraná, Departamento de Enfermagem, Paranavaí, PR, Brasil.; 2 Universidade Federal do Mato Grosso do Sul, Departamento de Enfermagem, Campo Grande, MS, Brasil.; 3 Universidade Federal de São Carlos, São Carlos, SP, Brasil.; 4 Escola Superior de Enfermagem do Porto, Porto, Portugal.

**Keywords:** Lúpus Eritematoso Sistêmico, Gravidez de Alto Risco, Doenças Autoimunes, Enfermagem, Teoria Fundamentada, Interacionismo Simbólico

## Abstract

**Objetivo:**

Construir uma teoria substantiva acerca dos significados atribuídos à vivência da gestação por mulheres com Lúpus Eritematoso Sistêmico.

**Métodos:**

Estudo qualitativo que utilizou como referencial teórico o Interacionismo Simbólico, e metodológico, a Teoria Fundamentada nos Dados, vertente construtivista. A coleta de dados ocorreu entre janeiro e agosto de 2022 mediante entrevista em profundidade, vídeo, audiogravada e realizada de forma remota. A amostragem teórica foi constituída por 27 participantes, distribuídos em quatro grupos amostrais.

**Resultados:**

Da análise comparativa constante construiu-se o fenômeno central: *Percebendo o planejamento reprodutivo como a chave do processo gestacional no Lúpus Eritematoso Sistêmico*, constituído por duas categorias, as quais mostram experiências e significados divergentes a partir do planejamento ou não da gestação. Considerações finais: A interação pessoal com o fenômeno, com base ou não nos “pilares” do planejamento reprodutivo, e a interação social com a equipe multiprofissional em saúde refletem no modo como a mulher percebe, comporta-se e significa a gestação. Assim, reitera-se que o planejamento reprodutivo é significado como a chave do desfecho gestacional no LES.

## INTRODUÇÃO

Considerado importante fator de risco durante o processo gravídico-puerperal, o Lúpus Eritematoso Sistêmico (LES) estratifica a gestação como de alto risco. Por isso, mulheres com essa doença precisam planejar a gestação e serem acompanhadas por uma equipe multiprofissional em saúde^(1,2,3)^, tendo em vista que a adequação medicamentosa, bem como o pré-natal, devem ser iniciados imediatamente^(4,5)^.

As manifestações do LES na gestação e a gestação na atividade da doença constituem temas amplamente discutidos na literatura^(6,7,8,9,10)^, cujos estudos evidenciaram aumento da atividade da doença durante a gestação e no período pós-parto^(8,9,10,11)^. No entanto, os riscos de exacerbações parecem ser maiores quando o LES se encontra em atividade no período de, pelo menos, seis meses anteriores à preconcepção. Da mesma forma, os riscos são reduzidos quando a concepção ocorre com a doença em remissão^(11)^.

A gestação, por ser de alto risco, é percebida pelas mulheres com LES como um processo complexo que desencadeia sentimentos de medo, angústias e incertezas^(2,3,8,10)^. Assim, esse fenômeno é significado a partir de experiências como presença de complicações pela ativação da doença, abortamento de repetição, partos prematuros, pré-eclâmpsia, má formação fetal, e complicações da Síndrome Antifosfolípide (SAF), as quais certamente impactam sobre a forma como as mulheres vivenciam essa experiência^(12)^. Não obstante, a assistência à saúde recebida, também, constitui parte desse processo^(1,13)^.

Ante isso, destaca-se a necessidade de compreender de forma aprofundada os significados atribuídos à gestação por mulheres com LES, considerando a sua interação pessoal com o fenômeno, bem como sua interação social com a equipe multiprofissional em saúde. Desse modo, definiu-se por objetivo construir uma teoria substantiva acerca dos significados atribuídos à vivência da gestação por mulheres com Lúpus Eritematoso Sistêmico.

## MÉTODO

### Desenho do Estudo/Referencial Teórico e Metodológico

Pesquisa explicativa, de natureza qualitativa, que teve como base teórico-conceitual o Interacionismo Simbólico (IS), o qual pressupõe que os significados são atribuídos a partir da experiência vivida^(14,15)^. Como referencial metodológico, adotou-se a Teoria Fundamentada nos Dados na perspectiva construtivista (TFDC), a qual considera que as experiências de pesquisador e participante diante do contexto são relevantes para a interpretação do fenômeno^(16)^. Na descrição dos resultados do estudo, utilizaram-se as diretrizes do Consolidated Criteria for Reporting Qualitative Research (COREQ)^(17)^.

### Local do Estudo

As mulheres com LES participantes do estudo foram localizadas no grupo privado “Lúpus Brasil o desabafo”, hospedado na rede social Facebook®, criado em 2014, que possui mais de 30 mil membros. O objetivo do grupo é favorecer a troca de informações e experiências, sendo a gestação tema recorrente de discussões.

### População

Participaram 27 mulheres, divididas em quatro grupos amostrais (GA). As integrantes foram convidadas mediante postagem pública no grupo “Lúpus Brasil o desabafo” e mensagens de texto, enviadas àquelas que participavam das publicações e faziam comentários sobre LES e gestação.

### Critérios de Seleção

Os critérios de inclusão foram: ter 18 anos ou mais, ter vivenciado a gestação com LES; e ter acesso à rede de internet com dispositivo tecnológico para vídeo chamada. O único critério de exclusão estabelecido foi: apresentar alguma dificuldade na comunicação, sendo uma mulher excluída por surdez.

### Definição da Amostra

Para a conformação do primeiro GA, considerou-se a hipótese: o significado atribuído à gestação está relacionado com a forma com que a mulher vivencia o fenômeno. Este GA foi conformado intencionalmente por 14 mulheres com LES que vivenciaram a gestação de alto risco. Durante a coleta e análise de dados, evidenciou-se que a experiência gestacional se difere e/ou se modifica quando a mulher experiencia outras condições clínicas associadas. Dentre estas, a SAF foi relatada com frequência, e isto parecia demarcar mudança no comportamento e delinear a percepção acerca da gestação.

Mediante esse dado, formulou-se a seguinte hipótese: o significado atribuído pode diferenciar-se à medida que a mulher vivencia outra condição clínica com potencial risco de complicações gestacionais. Desse modo, o segundo GA foi constituído por seis mulheres que tiveram trombofilia durante a gestação. Na análise dos dados desse grupo identificou-se que o risco de perda gestacional ou a vivência de aborto parecia influenciar sobre os significados da experiência, sendo levantada a seguinte hipótese: a forma como a mulher significa a gestação pode ser modificada quando esta vivencia perdas gestacionais.

Assim, o terceiro GA foi constituído por quatro mulheres com LES que sofreram abortamentos espontâneos. Dentre as participantes desse grupo havia uma mulher que vivenciou uma gestação na Itália, e assim surgiu a hipótese: engravidar com LES fora do país de origem pode modificar a forma como a mulher significa a gestação. Desse modo, o quarto GA foi constituído por três mulheres brasileiras que vivenciaram a gestação como imigrantes em outros países.

A constituição dos grupos amostrais finalizou-se a partir da saturação teórica dos dados, ou seja, as novas entrevistas e os novos dados coletados não traziam novos aprofundamentos ao fenômeno central construído, entendendo assim que o objetivo da investigação foi alcançado, finalizando o recrutamento de novas participantes.

### Período de Coleta de Dados

Os dados foram coletados entre janeiro e agosto de 2022, mediante entrevistas em profundidade, realizadas por vídeo chamada. Estas foram vídeo e áudio gravadas após autorização e tiveram duração média de setenta e cinco minutos. Os aplicativos *WhatsApp e Messenger* e a plataforma de comunicação por vídeo *Google Meet* foram utilizadas.

Em todas as entrevistas foi utilizada uma questão norteadora apropriada a cada GA, como segue: conte-me como foi a sua gestação com LES; conte-me como foi para você gestar com LES e trombofilia; conte-me como ocorreram seus abortos nas gestações com LES; conte-me como foi para você gestar com LES fora do seu país de origem. Todas as entrevistas foram realizadas por uma enfermeira, doutoranda em Enfermagem com experiência em pesquisa qualitativa e que vivenciou abortos espontâneos de repetição e uma gestação com trombofilia.

### Análise de Dados

Os dados foram organizados e analisados no software MAXQDA *plus* 2020. As entrevistas foram transcritas e submetidas ao estudo dos textos, acompanhadas do desenvolvimento de memorandos e diagramas. A codificação inicial ocorreu palavra a palavra, linha a linha e incidente por incidente, mediante o método comparativo constante, gerando os códigos iniciais e provisórios. A codificação focalizada foi realizada, utilizando os códigos iniciais mais significativos e frequentes, comparando os códigos iniciais de uma mesma entrevista com os das entrevistas subsequentes, objetivando criar códigos mais direcionados, seletivos e conceituais. Isto permitiu a separação, classificação, integração e organização dos dados, visando à conceituação do material empírico em subcategorias e, posteriormente, a identificação das propriedades conceituais das categorias. A Figura 1 apresenta o processo de análise de dados, produção do fenômeno central, das categorias e subcategorias que compuseram a teoria substantiva.

**Figura 1:** Diagrama do Processo de análise de dados, produção da categoria central, das categorias e subcategorias – Maringá, PR, Brasil, 2023.

### Aspectos Éticos

O desenvolvimento do estudo foi aprovado pelo Comitê Permanente de Ética em Pesquisa com Seres Humanos da instituição signatária, parecer n^o^ 5.132.482, CAAE: 53038321.8.0000.0104, respeitando a Resolução n. º 466/12 e as orientações para pesquisa em ambiente virtual da Comissão Nacional de Ética em Pesquisa. O Termo de Consentimento Livre e Esclarecido (TCLE) foi assinado pelo *Google Forms.* Os extratos das falas foram identificados pela letra G referente ao grupo amostral e ao número respectivo, seguido da letra P (participante) e do número respectivo à ordem da entrevista. Exemplo: (G1, P3).

Os critérios de ajuste, compreensão, generalização e controle foram avaliados positivamente, sendo possível perceber que a representação diagramática da teoria foi satisfatória. *Consigo me ver nas duas situações onde na gestação que não planejei acabei tendo muitas complicações. Quando engravidei de forma planejada e fui acompanhada pelos profissionais tive uma gestação mais tranquila e consegui chegar até o final.* (P1). *O planejamento da gestação foi essencial para o nascimento do meu filho, evitou perdas e sofrimentos, consigo ver isso na teoria* (P4). *Olhando a teoria, é possível perceber que o mais importante é o planejamento da gestação. Foi assim comigo, a primeira eu perdi, depois conseguir ter meu filho* (P6).

No quesito controle, as avaliadoras relataram que a teoria revelava claramente a relação Lúpus e gestação. *Para quem vivenciou as duas experiências, a teoria faz sentido e apresenta o que acontece em uma gestação com Lúpus* (P1)*. É a verdade e a necessidade mostrada em uma figura, mostra a importância de uma gravidez planejada* (P2).

Depois desse processo, a teoria foi refinada em termos de aperfeiçoamento estético e termos técnicos, buscando mais consistência à sua apresentação.

### Validação da Teoria

Primeiramente, realizou-se uma validação interna, na qual a teoria foi comparada com os dados interpretados na etapa inicial, objetivando aperfeiçoar as interpretações e identificar se a teoria apresentada era explicativa aos achados. Posteriormente, a teoria foi apresentada, remotamente pelo *Google Forms,* a um grupo de seis avaliadoras, sendo todas participantes do estudo. A avaliação seguiu a estrutura preconizada por Strauss: Ajuste, Compreensão, Generalização e Controle. A validação ocorreu no mês de fevereiro de 2023.

## RESULTADOS

Participaram 27 mulheres com idades entre 24 e 46 anos, residentes em quatro regiões brasileiras, em 11 Estados e três em outros países (Alemanha, Japão e Suécia). O tempo de diagnóstico da doença variou de dois a 28 anos, e o número de gestações de um a seis, sendo cinco o número máximo de abortos. As principais comorbidades secundárias ao LES experienciadas foram: SAF (23), Artrite reumatoide (3), Fibromialgia (3), Anemia falciforme (1), Hipotireoidismo (1), Síndrome de Reynaud (1), Diabetes Mellitus (1) e Nefrite lúpica (1).

A Figura 2, apresenta o fenômeno central, *Percebendo o planejamento reprodutivo como a chave do processo gestacional no Lúpus Eritematoso Sistêmico,* constituído por duas categorias relacionadas, as quais retratam a experiência vivenciada na gestação planejada e na não planejada.

**Figura 2:** Diagrama de apresentação da teoria substantiva: Percebendo o planejamento reprodutivo como a chave do processo gestacional no Lúpus Eritematoso Sistêmico – Maringá, PR, Brasil (autores 2023).

### Percebendo o Planejamento Reprodutivo como a Chave do Processo Gestacional no Lúpus Eritematoso Sistêmico

O significado atribuído à gestação no contexto do LES se estabelece a partir da forma como o fenômeno se apresenta na vida da mulher, o que, por sua vez, é mediado pelos significados já atribuídos à doença e que se modifica com a experiência vivida, e a partir da interação social com o mundo ao redor. Desse modo, o significado atribuído à gestação por mulheres com a mesma condição crônica pode ser totalmente diverso, a depender do fato de a mesma ter sido ou não planejada.

### Vivenciando uma Gestação Planejada

A gestação planejada é possível quando a mulher tem conhecimento adequado sobre o Lúpus e suas implicações. Receber orientações pode impactar na construção do “Self social” de forma positiva e influenciar os comportamentos, a experiência e os significados atribuídos. *Receber orientações sobre a gestação foi fundamental, porque eu tive a oportunidade de me preparar para esse momento. Eu aprendi sobre a minha doença e o que era preciso fazer para ter uma boa gestação* (G1, P2)*. Desde menina meu reumatologista já me falava que eu teria que me cuidar e que não poderia engravidar sem planejar antes, eu cresci com isso na minha cabeça. A gestação não foi fácil, mas eu já sabia dos riscos e o que poderia acontecer e isso ajudou* (G2, P20).

Ao vivenciar o fenômeno de modo planejado, com apoio dos profissionais de saúde, a gestação é vivenciada com mais segurança e tranquilidade, uma vez que reduz os riscos de complicações e desfechos gestacionais desfavoráveis. *Durante toda a gestação, minha reumatologista e ginecologista estiveram comigo, cuidaram de mim, a gente até criou uma amizade de tanto que a gente se falava e isso ajudou muito, foi essencial* (G2, P15). *Na minha segunda gestação, eu tive um acompanhamento profissional, um reumatologista, uma obstetra de alto risco. Por conta disso, eu tive uma gestação mais tranquila, com menos complicações.* (G1, P14) *Depois de ter passado pelas duas experiências: uma gestação não planejada e outra planejada, eu posso dizer que me sinto vencedora, hoje eu entendo a importância que é você planejar uma gestação quando se tem Lúpus* (G1, P7)*. Ser gestante com Lúpus é ser guerreira para aguentar e conseguir chegar ao final. Porque é um dia de cada vez, a gente não sabe se no outro dia a criança ainda vai estar ali, então tem que ter Fé e muita força. Sentir-se apoiada pela sua equipe médica, pela sua família e realizar certinho o tratamento e os cuidados são muito importantes. Hoje eu acredito que a gestação é possível, não é fácil, mas possível. É preciso planejar* (G1, P12).

O planejamento reprodutivo oportuniza o alcance do sonho da maternidade e a decisão compartilhada entre a mulher e o profissional de saúde sobre o momento adequado para gestar mostra-se essencial. *Antes de engravidar novamente, eu planejei a gestação, junto com reumatologista e ginecologista, isso foi essencial. A gente se programou e eu só engravidei quando minha doença estava em remissão* (G2, P17). *A minha gestação foi toda planejada, eu já vinha me preparando para isso há muito tempo. Quando decidi procurei a equipe médica e fui muito bem assistida. Isso foi um dos pontos mais importantes* (G4, P 25). *Quando eu engravidei, meu Lúpus estava em remissão há mais de um ano, e isso é muito importante. Se quiser ter filhos e tem Lúpus tem que esperar a doença entrar em remissão. Meu médico me ajudou a decidir o momento certo de engravidar* (G2, P16).

Isso porque possibilita um preparo psicológico, em especial quando existe uma condição que exige tratamentos complexos. *Tratar da trombofilia não é fácil, ninguém gosta de injeção. Mas eu aceitei bem. Já sabia que quando ficasse grávida teria que tratar e isso me ajudou. A gente se sente vitoriosa, é uma superação muito grande passar por isso*. (G2, P19). *Aquelas injeções simbolizam o amor, a força e a coragem de uma mãe em busca do seu sonho. É um ato de amor se furar todos os dias. Ver seu corpo sangrar, os hematomas enormes que se formam. A gente deposita toda a nossa esperança naquela injeção* (G2 P15).

A assistência prestada por diferentes profissionais também foi valorizada por brasileiras que moram em outros países, demostrando que sentir-se apoiada é essencial. *Eu fiquei muito confiante, não senti medo, recebi muita informação. Tive muito apoio da minha equipe médica. Eu sabia que estava bem assistida, e isso fez diferença* (G4, P25)*. Eu realizei o meu pré-natal com uma equipe multiprofissional, eu a via praticamente todas as semanas, fazia exames, muito controle, e assim foi até o final da gestação. Eu criei um bom vínculo com a equipe, e isso foi essencial* (G4, P27).

Tais experiências, além de contribuírem para a construção de significados positivos, são responsáveis por proporcionarem uma nova percepção sobre os profissionais de saúde. *Desde menina, eu ouvia que jamais poderia engravidar, passei toda a minha adolescência ouvindo coisas negativas dos médicos, hoje a gente percebe que eles melhoraram. Já não se houve mais tantas vezes aquela coisa ruim, e isso é muito importante. Faz a gente se sentir melhor. É muito bom saber que os profissionais de hoje já não falam as mesmas coisas que os de antigamente* (G4, P27).

### Vivenciando os Dissabores de uma Gestação não Planejada

Ao vivenciar uma gestação não planejada e permeada por complicações, as mulheres atribuem significados negativos à experiência. *Para mim ser gestante com Lúpus é horrível. Me senti muito mal, muito deprimida, fiquei com vontade de desistir de tudo. Foi horrível* (G1, P9). *Eu não indico uma gestação para uma mulher com Lúpus, nunca na vida, foi a pior fase da minha vida. Achei que fosse morrer. Filhos nunca mais quero ter* (G4, P26).

Algumas mulheres com LES não acreditavam que pudessem engravidar e por esta razão não se preocuparam em fazer planejamento reprodutivo. *Eu passei a minha vida acreditando que nunca teria filhos. Que mulher com Lúpus não pode ter filhos. Quando descobri que estava grávida foi aquele susto, medo do que poderia acontecer comigo e com o bebê. Foi desesperador* (G2, P19).

Esta concepção é decorrente de orientações equivocadas recebidas ao longo da vida, inclusive de profissionais de saúde. *Eu cresci ouvindo que mulher com Lúpus não tem filhos, e isso me acompanhou por muito tempo. Quando conheci meu marido até disse para ele: olha tenho Lúpus nunca poderei te dar um filho. Eu acreditava nisso de verdade. Porque foi isso que os médicos me ensinaram a vida toda* (G4, P27). *Eu me sentia muito mal, sentimentos de incapacidade, de fracasso. É o sonho de qualquer mulher ser mãe. É muito difícil passar uma vida ouvindo que não pode ter filhos* (G3, P22).

A ausência de orientação profissional adequada, com destaque para o planejamento reprodutivo, constitui fonte de sentimentos de menos valia e desinformação. *Eu me sentia a pior mulher do mundo. Tentei tantas vezes e cada vez que perdia eu ouvia: você vai morrer, é mais fácil morrer do que conseguir ter um filho com Lúpus. Isso me machucava muito. Porque era o meu sonho ser mãe, eu me sentia muito triste, incapaz* (G3, P23). *Eu nunca tinha recebido orientações sobre gestação, o que eu sabia era o que eu busquei na internet. Médico nenhum me orientou sobre isso. Então, quando eu engravidei, tive muitas complicações, fui para UTI, quase morri e teria morrido se não tivesse tido a sorte de ser atendida em um bom hospital* (G1, P10). *Eu engravidei sem planejar. Eu não sabia muita coisa sobre a minha doença, nem sabia que tinha que planejar, e aconteceu tudo isso, perdi meu bebê, fiquei muito doente, sofri muito* (G3, P21).

A partir da perspectiva de não poder gestar, muitas mulheres não procuram o planejamento reprodutivo e acabam sofrendo complicações gestacionais que impactam negativamente em suas vidas e no significado atribuído. *Eu tive seis gestações e cinco abortos, um atrás do outro. Foi muito difícil passar por todas essas perdas. Como não tratei adequadamente nas outras gestações, perdi, e só na última com todos os tratamentos corretos deu certo* (G3, P 23). *Eu engravidei na primeira vez com doença em atividade, fiquei muito mal, o Lúpus piorou, fiquei internada a maior parte do tempo, tive um AVC, hipertensão, pré-eclâmpsia e perdi meu bebê. Foi um processo muito difícil* (G1, P12). *Meus dois filhos nasceram prematuros com 32 e 34 semanas, ficaram internados e eu quase morri* (G1, P11).

Doenças subjacentes como a SAF são vivenciadas, o que exige tratamentos complexos. *Tratar da SAF é muito difícil. Injeção todo dia. A gente fica toda roxa, dor, é muito sofrido* (G2 P18). *Não gosto nem de lembrar, foi a pior parte de tudo. Aquelas injeções machucam, sangram. Não sei se teria coragem de enfrenta-las novamente* (G4 P25). *Eu sempre tive pavor de agulhas, com certeza foi a parte mais difícil. Eu nunca tive coragem de aplicar em mim, sempre foi meu marido* (G2, 17).

As fragilidades na assistência também foram percebidas por mulheres que vivenciaram a gestação em outros países. *Aqui na Suécia, os médicos também são contra gestação em mulheres com Lúpus, ficam horrorizados quando uma lúpica engravida. A gente percebe que falta muito conhecimento. Eles não têm segurança para acompanhar uma gestação como essa* (G4, P 25)*. Aqui no Japão, o aborto é autorizado, tem Lúpus pode abortar. Eles são totalmente contra gestação em mulheres com Lúpus e não possuem preparo nenhum para atender. A minha experiência em ter tido filho aqui foi horrível, aterrorizante* (G4, P 26).

Os significados atribuídos à gestação não planejada são negativos e têm relação direta com o processo experenciado. *Foi uma gravidez turbulenta, muitas dores, muitos hospitais, internações até nascer de sete meses* (G1, P 11). *A experiência que eu vivi foi horrível, infelizmente. Não quero ficar grávida nunca mais na minha vida* (G4, P26).

## DISCUSSÃO

Os significados atribuídos à gestação são influenciados pelo planejamento reprodutivo ou sua ausência. Ademais, relacionam-se com o modo com que a mulher vivenciou o processo gestacional, sendo a interação pessoal e a interação social as bases estruturantes para a construção desses significados. Nesse sentido, os resultados corroboram as perspectivas do IS, as quais enfatizam que o significado é criado a partir do modo como os sujeitos processam as experiências, como o fenômeno se apresenta em sua vida e como os mesmos e seus reflexos são interpretados^(14,15)^.

Foi possível compreender que ao vivenciar uma gestação planejada com apoio de uma equipe multiprofissional em saúde, os significados atribuídos pelas mulheres a partir da interação do eu, da mente e da sociedade são positivos, no sentido de terem conseguido superar os desafios e as limitações do LES e das condições associadas. Isto ocorre porque essas mulheres vivenciaram uma gestação mais tranquila e saudável, e, ao mesmo tempo, sentiram-se apoiadas e seguras.

Uma coorte retrospectiva realizada em Campinas, São Paulo, Brasil, visando avaliar os efeitos da gravidez em pacientes com LES, evidenciou que, quando as mulheres realizam o planejamento reprodutivo e engravidam com a doença em remissão, o processo gestacional é vivenciado de forma mais suave, com menos riscos de complicações e com maiores chances de desfechos positivos^(12)^.

A literatura científica enfatiza a importância do planejamento reprodutivo^(12,18,19)^ e as implicações positivas em relação a esse processo, como: redução das complicações; diagnóstico e tratamento precoce de fatores de riscos e doenças subjacentes; e melhor preparo físico e psicológico, bem como possibilidade de planejamento do parto e puerpério^(12,18,19)^.

Quando existe vínculo e confiança entre a gestante e os profissionais que acompanham a gestação de alto risco, os desfechos obstétricos, bem como a condução do processo gestacional, são vivenciados de forma mais positiva^(9,13)^. Essa relação predispõe à tomada de decisões que favoreçam um atendimento especializado e humanizado, cujo objetivo é a segurança e a qualidade do cuidado, bem como o empoderamento das mulheres ante o período gestacional.

A interação entre a mulher e o profissional que acompanha sua condição de saúde possibilita que a gestação ocorra em momento oportuno, reduzindo fatores de riscos. Isso porque o sucesso da gestação, na maioria das vezes, está atrelado ao início da gestação em momento oportuno com doença em remissão por, no mínimo, seis meses de preconcepção^(10,13,18,19)^.

Desse modo, quando planejada, o fenômeno é vivido de modo positivo, pois, além de pessoalmente estar mais bem preparada, a mulher poderá contar com o apoio de uma equipe multiprofissional em saúde, o que contribui para a vivência de uma experiência mais saudável e, consequentemente, sua ressignificação. Segundo o IS, essa interação social, ocorrida ao longo do tempo, permite que a mulher assuma sua identidade, organize o seu comportamento, posicione-se ante a condição clínica e simbolize suas experiências. Assim, a interação social é entendida como uma das principais ferramentas de apoio ao desfecho gestacional e, também, da criação de significados sobre o processo vivenciado^(14,15)^.

No entanto, na maioria das vezes, a gestação em mulheres com LES ocorre sem planejamento reprodutivo, podendo desencadear experiências complexas, permeadas por complicações^(1,2,3)^. Isto ocorre porque passaram parte da vida acreditando que não poderiam engravidar ou ainda por não receberem orientações adequadas por parte dos profissionais. Deste modo, conceitos, comportamentos e atitudes são estabelecidos a partir desses ensinamentos, resultando em gestações não planejadas e não acompanhadas por profissionais de saúde especializados.

Houve um período em que a orientação médica era a de que mulheres com LES deveriam evitar a gestação, uma vez que essa representava grande risco de complicações maternas e fetais. No entanto, com o avanço da ciência, da capacitação profissional, da maior acuidade diagnóstica e dos recursos terapêuticos, atualmente a gestação em mulheres com LES é totalmente viável^(12,18,19)^. Contudo, são consideradas gestações de alto risco, e requerem, além de um acompanhamento diferenciado, a existência de vínculo e confiança entre paciente e profissionais de saúde^(12,18)^.

Algumas mulheres do estudo, no entanto, relataram fragilidades na interação social com os profissionais, perfazendo a ideia de que a gestação ocorreu sem planejamento por falta de orientação e conhecimento específico. Outros estudos já fizeram referência à precariedade da assistência ofertada às mulheres com LES, com destaque para a falta de orientação sobre planejamento reprodutivo e pessimismo profissional referente à combinação LES e gestação^(1,13)^. Por vezes, essa precariedade assistencial é decorrente do enfoque profissional no tratamento medicamentoso da doença, tornando os desejos, sonhos e ideais dessas mulheres invisíveis.

Essas características assistenciais impactam negativamente na ocorrência de gestações não planejadas e experiências traumáticas^(1,2,3)^. Além disso, na presença de SAF, o tratamento torna-se ainda mais complexo, requerendo aplicações diárias de anticoagulantes injetáveis, os quais desencadeiam dor, hematomas e sangramentos^(20)^, contribuindo para uma percepção negativa da experiência.

Assim, a construção do “Self” a partir dessa vivência é, em sua maioria, negativa, visto que o processo é vivenciado de forma peculiar, permeado por sentimentos de medo, angústias e incertezas. A falta de interação social ou a interação social prejudicada com a equipe multiprofissional, produz impactos ainda mais negativos. Assim, torna-se importante, proporcionar visibilidade à mulher e seus anseios ao longo do acompanhamento, de modo a compreender que os desejos/sonhos podem se alterar a depender do estágio que a doença se encontra e dos fatores de risco envolvidos.

É importante destacar que a vigilância de uma mulher com LES deve ser realizada do período preconcepção até o puerpério^(5,21)^, com o objetivo de investigar precocemente fatores de riscos e doenças que possam impactar nos desfechos gestacionais^(5,13,22)^. O aconselhamento preconcepção necessita incluir orientações como: risco de exacerbação da doença e as potenciais complicações obstétricas, fetais e perinatais associadas a gestação não planejada; riscos de prematuridade; investigação de situações clínicas precoces; a importância de gestação com a doença em remissão, bem como a importância de acompanhamento pré-natal de alto risco^(5)^.

Com o avanço da ciência e do conhecimento científico sobre esse fenômeno, o planejamento reprodutivo ganha cada vez mais destaque e indicação, devendo esse ser identificado como uma das formas de diminuir a morbimortalidade e aumentar o sucesso destas gestações^(9)^. Assim, o planejamento da gestação com LES e a vigilância reprodutiva da mulher potencializam as chances de bons desfechos gestacionais, contribuindo para a construção de significados positivos.

Uma possível limitação do estudo está relacionada ao viés de seleção, visto que as mulheres com LES foram localizadas em uma plataforma virtual, o que limitou a participação daquelas que não tinham acesso à internet. Entretanto, os resultados desse estudo favorecem o conhecimento científico acerca da gestação em mulheres com LES e gera subsídios para discussões acerca da assistência à saúde voltada a esse público, bem como para a compreensão sobre a importância do planejamento reprodutivo para gestações com essa condição crônica. Sugere-se que pesquisas futuras investiguem o papel da família ante esse fenômeno.

## CONSIDERAÇÕES FINAIS

A construção dessa teoria substantiva concluiu que os significados atribuídos à gestação por mulheres com LES diferenciam-se a partir da experiência vivida, a qual por sua vez é influenciada pelo planejamento ou não da gravidez. A vivência de uma gestação planejada com apoio multiprofissional em saúde é significada como positiva, uma vez que oportuniza desfechos gestacionais favoráveis. Em contrapartida, a gestação não planejada e vivenciada sem apoio multiprofissional ou com apoio fragilizado é significada negativamente, em especial pela vivencia de complicações, construção de sentimentos negativos e desfechos gestacionais desfavoráveis.

A interação pessoal com o fenômeno é diretamente afetada pela situação da mulher com LES ter planejado ou não sua gestação e da interação social estabelecida com a equipe multiprofissional em saúde em seu acompanhamento no processo saúde/doença/gestação. Essas influenciam diretamente na maneira como a mulher percebe, comporta-se e significa a gestação. Assim, o planejamento reprodutivo é percebido como a chave do desfecho gestacional no LES.

## Data Availability

Todo o conjunto de dados que dá suporte aos resultados deste estudo foi publicado no próprio artigo.

# Translation: Significados atribuidos a la gravidez por mujeres con Lupus Eritematoso Sistémico: una teoría basada en datos

**DOI:** 10.1590/1980-220X-REEUSP-2024-0413es

**Authors:** Rebeca Rosa de Souza, Mayckel da Silva Barreto, Elen Ferraz Teston, Monika Wernet, Maria Eduarda Pascoaloto da Silva, Alexandrina Maria Ramos Cardoso, Sonia Silva Marcon

**Affiliations:** 1 Universidade Estadual do Paraná, Departamento de Enfermagem, Paranavaí, PR, Brasil.; 2 Universidade Federal do Mato Grosso do Sul, Departamento de Enfermagem, Campo Grande, MS, Brasil.; 3 Universidade Federal de São Carlos, São Carlos, SP, Brasil.; 4 Escola Superior de Enfermagem do Porto, Porto, Portugal.

**Keywords:** Lupus Eritematoso Sistémico, Embarazo de Alto Riesgo, Enfermedades Autoinmunes, Enfermería, Teoría Fundamentada, Interaccionismo Simbólico

## Abstract

**Objetivo:**

Construir una teoría sustantiva sobre los significados atribuidos a la vivencia del embarazo por mujeres con Lupus Eritematoso Sistémico.

**Métodos:**

Estudio cualitativo que utilizó como marco teórico el Interaccionismo Simbólico y como marco metodológico la Teoría Fundamentada, enfoque constructivista. La recolección de datos se realizó entre enero y agosto de 2022 mediante entrevistas en profundidad, grabadas en video, audio y realizadas de forma remota. La muestra teórica estuvo compuesta por 27 participantes, distribuidos en cuatro grupos muestrales.

**Resultados:**

A partir del análisis comparativo constante se construyó el fenómeno central: *Entendiendo la planificación reproductiva como clave del proceso gestacional en el Lupus Eritematoso Sistémico*, compuesta por dos categorías, que muestran experiencias y significados divergentes en función de si el embarazo fue planeado o no.

**Consideraciones finales:**

La interacción personal con el fenómeno, basada o no en los “pilares” de la planificación reproductiva, y la interacción social con el equipo multidisciplinario de salud reflexionan sobre el modo en que la mujer percibe, se comporta y da sentido al embarazo. Por tanto, se reitera que la planificación reproductiva se considera clave para el resultado gestacional en el LES.

## INTRODUCCIÓN

Considerado un factor de riesgo importante durante el proceso gravídico-puerperal, el lupus eritematoso sistémico (LES) clasifica el embarazo como de alto riesgo. Por ello, las mujeres con esta enfermedad deben planificar el embarazo y ser atendidas por un equipo multiprofesional en salud^(1,2,3)^, ya que la medicación adecuada, así como la atención prenatal, deben iniciarse de inmediato^(4,5)^. Las manifestaciones del LES en la gestación y la gestación en la actividad de la enfermedad son temas ampliamente discutidos en la literatura^(6,7,8,9,10)^, cuyos estudios han evidenciado un aumento de la actividad de la enfermedad durante la gestación y en el posparto^(8,9,10,11)^. Sin embargo, los riesgos de exacerbaciones parecen ser mayores cuando el LES se encuentra en actividad durante al menos seis meses antes de la preconcepción. Del mismo modo, los riesgos se reducen cuando la concepción se produce con la enfermedad en remisión^(11)^.

Al ser de alto riesgo, la gestación es percibida por las mujeres con LES como un proceso complejo que desencadena sentimientos de miedo, angustia e incertidumbre^(2,3,8,10)^. Así, este fenómeno se manifiesta a partir de experiencias como la presencia de complicaciones por la activación de la enfermedad, abortos recurrentes, partos prematuros, preeclampsia, malformaciones fetales y complicaciones del síndrome antifosfolípido (SAF), que sin duda influyen en la forma en que las mujeres viven esta experiencia^(12)^. No obstante, la asistencia sanitaria recibida también forma parte de este proceso^(1,13)^. Ante esto, destaca la necesidad de comprender en profundidad los significados atribuidos al embarazo por las mujeres con LES, considerando su interacción personal con el fenómeno, así como su interacción social con el equipo multiprofesional en salud. De este modo, se definió como objetivo construir una teoría sustantiva sobre los significados atribuidos a la vivencia del embarazo por las mujeres con Lupus Eritematoso Sistémico.

## MÉTODO

### Diseño del Estudio/Referencial Teórico y Metodológico

Investigación explicativa, de naturaleza cualitativa, que tuvo como base teórico-conceptual el Interaccionismo Simbólico (IS), el cual presupone que los significados se atribuyen a partir de la experiencia vivida^(14,15)^. Como referencia metodológica, se adoptó la Teoría Fundamentada en los Datos desde la perspectiva constructivista (TFDC), que considera que las experiencias del investigador y del participante ante el contexto son relevantes para la interpretación del fenómeno^(16)^. En la descripción de los resultados del estudio, se utilizaron las directrices de los Criterios Consolidados para la Presentación de Informes de Investigación Cualitativa (COREQ)^(17)^.

### Lugar del Estudio

Las mujeres con LES participantes en el estudio fueron localizadas en el grupo privado «*Lupus Brasil o desabafo*» (Lupus Brasil el desahogo), alojado en la red social Facebook®, creado en 2014, que cuenta con más de 30 000 miembros. El objetivo del grupo es favorecer el intercambio de información y experiencias, siendo la gestación un tema recurrente en los debates.

### Población

Participaron 27 mujeres, divididas en cuatro grupos muestrales (GM). Las integrantes fueron invitadas mediante una publicación pública en el grupo «Lupus Brasil o desabafo» y mensajes de texto enviados a aquellas que participaban en las publicaciones y hacían comentarios sobre el LES y el embarazo.

### Criterios de Selección

Los criterios de inclusión fueron: tener 18 años o más, haber vivido la gestación con LES y tener acceso a Internet con un dispositivo tecnológico para videollamadas. El único criterio de exclusión establecido fue: presentar alguna dificultad en la comunicación, por lo que se excluyó a una mujer por sordera.

### Definición de la Muestra

Para la conformación del primer GM, se consideró la hipótesis: el significado atribuido al embarazo está relacionado con la forma en que la mujer vive el fenómeno. Este GM fue conformado intencionalmente por 14 mujeres con LES que vivieron un embarazo de alto riesgo. Durante la recolección y el análisis de los datos, se evidenció que la experiencia gestacional difiere y/o se modifica cuando la mujer experimenta otras condiciones clínicas asociadas. Entre estas, el SAF se reportó con frecuencia, y esto parecía marcar un cambio en el comportamiento y delinear la percepción sobre la gestación.

A partir de este dato, se formuló la siguiente hipótesis: el significado atribuido puede diferir a medida que la mujer experimenta otra condición clínica con riesgo potencial de complicaciones gestacionales. De este modo, el segundo GM estuvo formado por seis mujeres que tuvieron trombofilia durante el embarazo. En el análisis de los datos de este grupo se identificó que el riesgo de pérdida gestacional o la experiencia del aborto parecían influir en los significados de la experiencia, por lo que se planteó la siguiente hipótesis: la forma en que la mujer significa el embarazo puede modificarse cuando experimenta pérdidas gestacionales.

Así, el tercer GM estuvo constituido por cuatro mujeres con LES que sufrieron abortos espontáneos. Entre las participantes de este grupo había una mujer que había vivido un embarazo en Italia, lo que dio lugar a la hipótesis: quedarse embarazada con LES fuera del país de origen puede modificar la forma en que la mujer significa el embarazo. Así, el cuarto GM estuvo formado por tres mujeres brasileñas que vivieron el embarazo como inmigrantes en otros países.

La constitución de los grupos muestrales finalizó a partir de la saturación teórica de los datos, es decir, las nuevas entrevistas y los nuevos datos recopilados no aportaban nuevos profundizaciones al fenómeno central construido, entendiendo así que el objetivo de la investigación se había alcanzado, finalizando el reclutamiento de nuevas participantes.

### Período de Recolección de Datos

Los datos se recopilaron entre enero y agosto de 2022, mediante entrevistas en profundidad, realizadas por videollamada. Estas fueron grabadas en vídeo y audio tras obtener la autorización y tuvieron una duración media de setenta y cinco minutos. Se utilizaron las aplicaciones *WhatsApp y Messenger* y la plataforma de comunicación por vídeo *Google Meet*.

En todas las entrevistas se utilizó una pregunta orientadora adecuada a cada GM, como sigue: cuénteme cómo fue su embarazo con LES; cuénteme cómo fue para usted estar embarazada con LES y trombofilia; cuénteme cómo fueron sus abortos en los embarazos con LES; cuénteme cómo fue para usted estar embarazada con LES fuera de su país de origen. Todas las entrevistas fueron realizadas por una enfermera, doctora en Enfermería con experiencia en investigación cualitativa y que ha sufrido abortos espontáneos recurrentes y un embarazo con trombofilia.

### Análisis de Datos

Los datos se organizaron y analizaron con el software MAXQDA *plus* 2020. Las entrevistas se transcribieron y se sometieron al estudio de los textos, acompañadas del desarrollo de memorandos y diagramas. La codificación inicial se realizó palabra por palabra, línea por línea e incidente por incidente, mediante el método comparativo constante, generando los códigos iniciales y provisionales. Se realizó una codificación focalizada, utilizando los códigos iniciales más significativos y frecuentes, comparando los códigos iniciales de una misma entrevista con los de las entrevistas posteriores, con el objetivo de crear códigos más específicos, selectivos y conceptuales. Esto permitió la separación, clasificación, integración y organización de los datos, con el fin de conceptualizar el material empírico en subcategorías y, posteriormente, identificar las propiedades conceptuales de las categorías. La Figura 1 presenta el proceso de análisis de datos, producción del fenómeno central, de las categorías y subcategorías que componen la teoría sustantiva.

**Figura 1:** Diagrama del proceso de análisis de datos, producción de la categoría central, de las categorías y subcategorías – Maringá, PR, Brasil, 2023.

### Aspectos Éticos

El desarrollo del estudio fue aprobado por el Comité Permanente de Ética en Investigación con Seres Humanos de la institución signataria, dictamen n.º 5.132.482, CAAE: 53038321.8.0000.0104, respetando la Resolución n.º 466/12 y las directrices para la investigación en entornos virtuales de la Comisión Nacional de Ética en Investigación. El Término de Consentimiento Libre y Esclarecido (TCLE) fue firmado por *Google Forms.* Los extractos de las declaraciones fueron identificados con la letra G correspondiente al grupo muestral y al número respectivo, seguida de la letra P (participante) y del número correspondiente al orden de la entrevista. Ejemplo: (G1, P3).

Los criterios de ajuste, comprensión, generalización y control se evaluaron positivamente, pudiéndose observar que la representación diagramática de la teoría fue satisfactoria. *Me veo en las dos situaciones en las que, en los embarazos que no planeé, acabé teniendo muchas complicaciones. Cuando me quedé embarazada de forma planificada y fui seguida por profesionales, tuve un embarazo más tranquilo y conseguí llegar hasta el final.* (P1). *La planificación del embarazo fue esencial para el nacimiento de mi hijo, evitó pérdidas y sufrimientos, puedo verlo en la teoría* (P4). *Al observar la teoría, se puede percibir que lo más importante es la planificación del embarazo. Así fue en mi caso, perdí el primero, pero luego pude tener a mi hijo* (P6).

En cuanto al control, las evaluadoras informaron que la teoría revelaba claramente la relación entre el lupus y el embarazo. *Para quienes han vivido ambas experiencias, la teoría tiene sentido y presenta lo que sucede en un embarazo con lupus* (P1)*. Es la verdad y la necesidad mostrada en una imagen, muestra la importancia de un embarazo planificado*(P2).

Tras este proceso, la teoría se perfeccionó en términos de estética y términos técnicos, buscando una mayor coherencia en su presentación.

### Validación de la Teoría

En primer lugar, se llevó a cabo una validación interna, en la que se comparó la teoría con los datos interpretados en la etapa inicial, con el objetivo de perfeccionar las interpretaciones e identificar si la teoría presentada era explicativa de los hallazgos. Posteriormente, la teoría se presentó, de forma remota a través de *Google Forms,* a un grupo de seis evaluadoras, todas ellas participantes en el estudio. La evaluación siguió la estructura recomendada por Strauss: Ajuste, Comprensión, Generalización y Control. La validación tuvo lugar en febrero de 2023.

## RESULTADOS

Participaron 27 mujeres de entre 24 y 46 años, residentes en cuatro regiones brasileñas, en 11 estados y tres en otros países (Alemania, Japón y Suecia). El tiempo de diagnóstico de la enfermedad varió de dos a 28 años, y el número de embarazos de uno a seis, siendo cinco el número máximo de abortos. Las principales comorbilidades secundarias al LES experimentadas fueron: SAF (23), artritis reumatoide (3), fibromialgia (3), anemia falciforme (1), hipotiroidismo (1), síndrome de Raynaud (1), diabetes mellitus (1) y nefritis lúpica (1).

La Figura 2 presenta el fenómeno central, *Percibir la planificación reproductiva como la clave del proceso gestacional en el lupus eritematoso sistémico,* constituido por dos categorías relacionadas, que reflejan la experiencia vivida en la gestación planificada y en la no planificada.

**Figura 2:** Diagrama de apresentação da teoria substantiva: Percebendo o planejamento reprodutivo como a chave do processo gestacional no Lúpus Eritematoso Sistêmico – Maringá, PR, Brasil (autores 2023).

### Percibir la Planificación Reproductiva Como Clave del Proceso Gestacional en el Lupus Eritematoso Sistémico

El significado que se le da al embarazo en el contexto del LES se basa en cómo se presenta el fenómeno en la vida de la mujer, que, a su vez, está mediado por los significados que ya se le han dado a la enfermedad y que cambian con la experiencia vivida y la interacción social con el mundo que la rodea. De este modo, el significado atribuido al embarazo por mujeres con la misma enfermedad crónica puede ser totalmente diferente, dependiendo de si ha sido planificado o no.

### Vivir una Gestación Planificada

La gestación planificada es posible cuando la mujer tiene un conocimiento adecuado sobre el lupus y sus implicaciones. Recibir orientación puede influir positivamente en la construcción del «yo social» e influir en los comportamientos, la experiencia y los significados atribuidos. *Recibir orientación sobre la gestación fue fundamental, porque tuve la oportunidad de prepararme para ese momento. Aprendí sobre mi enfermedad y lo que tenía que hacer para tener un buen embarazo (G1, P2). Desde niña, mi reumatólogo ya me decía que tenía que cuidarme y que no podía quedarme embarazada sin planificarlo antes, crecí con eso en mi cabeza. El embarazo no fue fácil, pero ya conocía los riesgos y lo que podía pasar, y eso me ayudó* (G2, P20).

Al vivir el fenómeno de forma planificada, con el apoyo de los profesionales de la salud, el embarazo se vive con más seguridad y tranquilidad, ya que se reducen los riesgos de complicaciones y desenlaces gestacionales desfavorables. *Durante todo el embarazo, mi reumatóloga y mi ginecóloga estuvieron conmigo, me cuidaron, incluso llegamos a hacer amistad porque hablábamos mucho y eso ayudó mucho, fue esencial* (G2, P15). *En mi segundo embarazo, tuve seguimiento profesional, un reumatólogo y una obstetra de alto riesgo. Gracias a ello, tuve un embarazo más tranquilo, con menos complicaciones.* (G1, P14) *Después de haber pasado por las dos experiencias: un embarazo no planificado y otro planificado, puedo decir que me siento ganadora, hoy entiendo la importancia de planificar un embarazo cuando se tiene lupus* (G1, P7)*. Estar embarazada con lupus es ser una guerrera para aguantar y llegar hasta el final. Porque es un día a la vez, no sabemos si al día siguiente el niño seguirá ahí, así que hay que tener fe y mucha fuerza. Sentirse apoyada por tu equipo médico, por tu familia y seguir al pie de la letra el tratamiento y los cuidados es muy importante. Hoy creo que el embarazo es posible, no es fácil, pero es posible. Hay que planificar* (G1, P12).

La planificación reproductiva permite alcanzar el sueño de la maternidad y la decisión compartida entre la mujer y el profesional de la salud sobre el momento adecuado para gestar resulta esencial. *Antes de volver a quedarme embarazada, planifiqué el embarazo junto con el reumatólogo y el ginecólogo, eso fue esencial. Nos programamos y solo quedé embarazada cuando mi enfermedad estaba en remisión* (G2, P17). *Mi embarazo fue totalmente planificado, llevaba mucho tiempo preparándome para ello. Cuando lo decidí, acudí al equipo médico y me atendieron muy bien. Ese fue uno de los puntos más importantes* (G4, P 25). *Cuando me quedé embarazada, mi lupus llevaba más de un año en remisión, y eso es muy importante. Si quieres tener hijos y tienes lupus, tienes que esperar a que la enfermedad entre en remisión. Mi médico me ayudó a decidir el momento adecuado para quedarme embarazada* (G2, P16).

Esto es porque permite una preparación psicológica, especialmente cuando existe una afección que requiere tratamientos complejos. *Tratar la trombofilia no es fácil, a nadie le gustan las inyecciones. Pero lo acepté bien. Ya sabía que cuando quedara embarazada tendría que tratarme y eso me ayudó. Uno se siente victorioso, es una gran superación pasar por esto*. (G2, P19). *Esas inyecciones simbolizan el amor, la fuerza y el coraje de una madre en busca de su sueño. Es un acto de amor pincharse todos los días. Ver tu cuerpo sangrar, los enormes hematomas que se forman. Ponemos toda nuestra esperanza en esa inyección* (G2 P15).

La asistencia prestada por diferentes profesionales también fue valorada por las brasileñas que viven en otros países, lo que demuestra que sentirse apoyada es esencial. «Me sentí muy segura, no sentí miedo, recibí mucha información. Tuve mucho apoyo de mi equipo médico. Sabía que estaba bien atendida y eso marcó la diferencia» (G4, P25)*. Realicé mi prenatal con un equipo multidisciplinar, lo veía prácticamente todas las semanas, me hacían pruebas, mucho control, y así fue hasta el final del embarazo. Creé un buen vínculo con el equipo, y eso fue esencial* (G4, P27).

Estas experiencias, además de contribuir a la construcción de significados positivos, son responsables de proporcionar una nueva percepción sobre los profesionales de la salud. *Desde niña, me decían que nunca podría quedarme embarazada, pasé toda mi adolescencia escuchando cosas negativas de los médicos, hoy nos damos cuenta de que han mejorado. Ya no se oyen tantas cosas malas, y eso es muy importante. Nos hace sentir mejor. Es muy bueno saber que los profesionales de hoy ya no dicen las mismas cosas que los de antes* (G4, P27).

### Vivenciar las Dificultades de un Embarazo no Planificado

Al vivir un embarazo no planificado y lleno de complicaciones, las mujeres atribuyen significados negativos a la experiencia. *Para mí, estar embarazada con lupus es horrible. Me sentí muy mal, muy deprimida, con ganas de dejarlo todo. Fue horrible* (G1, P9). *No recomiendo el embarazo a una mujer con lupus, nunca en la vida, fue la peor etapa de mi vida. Pensé que iba a morir. No quiero volver a tener hijos* (G4, P26).

Algunas mujeres con LES no creían que pudieran quedarse embarazadas y, por esta razón, no se preocuparon por planificar su reproducción. «Pasé mi vida creyendo que nunca tendría hijos. Que las mujeres con lupus no pueden tener hijos. Cuando descubrí que estaba embarazada, me asusté mucho, tenía miedo de lo que pudiera pasarme a mí y al bebé. Fue desesperante» (G2, P19).

Esta idea es consecuencia de orientaciones erróneas recibidas a lo largo de la vida, incluso por parte de profesionales de la salud. *Crecí escuchando que las mujeres con lupus no tienen hijos, y eso me acompañó durante mucho tiempo. Cuando conocí a mi marido, incluso le dije: mira, tengo lupus, nunca podré darte un hijo. Lo creía de verdad. Porque eso es lo que los médicos me enseñaron toda la vida* (G4, P27). *Me sentía muy mal, con sentimientos de incapacidad, de fracaso. Ser madre es el sueño de cualquier mujer. Es muy difícil pasar toda la vida escuchando que no puedes tener hijos* (G3, P22).

La falta de orientación profesional adecuada, especialmente en materia de planificación reproductiva, es fuente de sentimientos de inferioridad y desinformación. *Me sentía la peor mujer del mundo. Lo intenté tantas veces y cada vez que lo perdía oía: vas a morir, es más fácil morir que tener un hijo con lupus. Eso me dolía mucho. Porque mi sueño era ser madre, me sentía muy triste, incapaz* (G3, P23). Nunca había recibido orientación sobre el embarazo, lo que sabía era lo que había buscado en Internet. Ningún médico me orientó al respecto. Entonces, cuando quedé embarazada, tuve muchas complicaciones, fui a la UCI, casi muero y habría muerto si no hubiera tenido la suerte de ser atendida en un buen hospital (G1, P10). Quedé embarazada sin planearlo. *No sabía mucho sobre mi enfermedad, ni sabía que tenía que planificar, y pasó todo esto, perdí a mi bebé, me puse muy enferma, sufrí mucho* (G3, P21).

Desde la perspectiva de no poder gestar, muchas mujeres no buscan la planificación reproductiva y acaban sufriendo complicaciones gestacionales que repercuten negativamente en sus vidas y en el significado que les atribuyen. *Tuve seis embarazos y cinco abortos, uno tras otro. Fue muy difícil pasar por todas esas pérdidas. Como no me traté adecuadamente en los otros embarazos, los perdí, y solo en el último, con todos los tratamientos correctos, salió bien* (G3, P 23). *Me quedé embarazada la primera vez con la enfermedad activa, me encontré muy mal, el lupus empeoró, estuve ingresada la mayor parte del tiempo, tuve un ictus, hipertensión, preeclampsia y perdí a mi bebé. Fue un proceso muy difícil* (G1, P12). *Mis dos hijos nacieron prematuros, con 32 y 34 semanas, estuvieron hospitalizados y yo casi muero* (G1, P11).

Se padecen enfermedades subyacentes como la SAF, que requieren tratamientos complejos. *Tratar la SAF es muy difícil. Inyecciones todos los días. Nos quedamos todas moradas, con dolor, es muy doloroso* (G2 P18). *Ni siquiera me gusta recordarlo, fue lo peor de todo. Esas inyecciones duelen, sangran. No sé si tendría valor para volver a enfrentarlas* (G4 P25). *Siempre he tenido pánico a las agujas, sin duda fue lo más difícil. Nunca me atreví a ponérmelas, siempre lo hacía mi marido* (G2, 17).

Las deficiencias en la asistencia también fueron percibidas por mujeres que vivieron el embarazo en otros países. *Aquí en Suecia, los médicos también están en contra del embarazo en mujeres con lupus, se horrorizan cuando una mujer con lupus se queda embarazada. Nos damos cuenta de que hay mucha falta de conocimiento. No se sienten seguros para seguir un embarazo como este* (G4, P 25)*. Aquí en Japón, el aborto está permitido, las mujeres con lupus pueden abortar. Están totalmente en contra del embarazo en mujeres con lupus y no tienen ninguna preparación para atenderlas. Mi experiencia al tener un hijo aquí fue horrible, aterradora* (G4, P 26).

Los significados atribuidos al embarazo no planificado son negativos y están directamente relacionados con el proceso vivido. *Fue un embarazo turbulento, con mucho dolor, muchos hospitales, ingresos hasta que nació a los siete meses* (G1, P 11). *La experiencia que viví fue horrible, por desgracia. No quiero volver a quedarme embarazada en mi vida* (G4, P26).

## DISCUSIÓN

Los significados atribuidos al embarazo están influenciados por la planificación reproductiva o la ausencia de ella. Además, están relacionados con la forma en que la mujer vivió el proceso gestacional, siendo la interacción personal y la interacción social las bases estructurales para la construcción de estos significados. En este sentido, los resultados corroboran las perspectivas del SI, que enfatizan que el significado se crea a partir de la forma en que los sujetos procesan las experiencias, cómo se presenta el fenómeno en su vida y cómo ellos mismos y sus reflejos son interpretados^(14,15)^. Se pudo comprender que al vivir una gestación planificada con el apoyo de un equipo multiprofesional en salud, los significados atribuidos por las mujeres a partir de la interacción del yo, la mente y la sociedad son positivos, en el sentido de que lograron superar los desafíos y las limitaciones del LES y las condiciones asociadas. Esto se debe a que estas mujeres vivieron un embarazo más tranquilo y saludable y, al mismo tiempo, se sintieron apoyadas y seguras.

Una cohorte retrospectiva realizada en Campinas, São Paulo, Brasil, con el objetivo de evaluar los efectos del embarazo en pacientes con LES, evidenció que, cuando las mujeres planifican su reproducción y quedan embarazadas con la enfermedad en remisión, el proceso gestacional se vive de forma más suave, con menos riesgos de complicaciones y con mayores posibilidades de resultados positivos^(12)^. La literatura científica enfatiza la importancia de la planificación reproductiva^(12,18,19)^ y las implicaciones positivas de este proceso, tales como: reducción de complicaciones; diagnóstico y tratamiento precoz de factores de riesgo y enfermedades subyacentes; y mejor preparación física y psicológica, así como la posibilidad de planificar el parto y el puerperio^(12,18,19)^.

Cuando existe un vínculo y confianza entre la gestante y los profesionales que acompañan la gestación de alto riesgo, los desenlaces obstétricos, así como la conducción del proceso gestacional, se viven de forma más positiva^(9,13)^. Esta relación predispone a la toma de decisiones que favorecen una atención especializada y humanizada, cuyo objetivo es la seguridad y la calidad de la atención, así como el empoderamiento de las mujeres ante el período gestacional.

La interacción entre la mujer y el profesional que acompaña su estado de salud permite que la gestación se produzca en el momento oportuno, reduciendo los factores de riesgo. Esto se debe a que el éxito de la gestación, en la mayoría de los casos, está ligado al inicio de la gestación en el momento oportuno, con la enfermedad en remisión durante al menos seis meses antes de la preconcepción^(10,13,18,19)^.

De este modo, cuando se planifica, el fenómeno se vive de manera positiva, ya que, además de estar mejor preparada personalmente, la mujer puede contar con el apoyo de un equipo multidisciplinar en salud, lo que contribuye a una experiencia más saludable y, en consecuencia, a su resignificación. Según el IS, esta interacción social, que se produce a lo largo del tiempo, permite a la mujer asumir su identidad, organizar su comportamiento, posicionarse ante la condición clínica y simbolizar sus experiencias. Así, la interacción social se entiende como una de las principales herramientas de apoyo al desenlace gestacional y también a la creación de significados sobre el proceso vivido^(14,15)^. Sin embargo, en la mayoría de los casos, el embarazo en mujeres con LES se produce sin planificación reproductiva, lo que puede desencadenar experiencias complejas, plagadas de complicaciones^(1,2,3)^. Esto se debe a que han pasado parte de su vida creyendo que no podían quedarse embarazadas o a que no han recibido la orientación adecuada por parte de los profesionales. De este modo, se establecen conceptos, comportamientos y actitudes a partir de estas enseñanzas, lo que da lugar a embarazos no planificados y no acompañados por profesionales de la salud especializados.

Hubo un período en el que la orientación médica era que las mujeres con LES debían evitar el embarazo, ya que este representaba un gran riesgo de complicaciones maternas y fetales. Sin embargo, con los avances de la ciencia, la formación profesional, la mayor precisión diagnóstica y los recursos terapéuticos, actualmente el embarazo en mujeres con LES es totalmente viable^(12,18,19)^. No obstante, se consideran embarazos de alto riesgo y requieren, además de un seguimiento diferenciado, la existencia de un vínculo y confianza entre la paciente y los profesionales sanitarios^(12,18)^.

Algunas mujeres del estudio, sin embargo, informaron de fragilidades en la interacción social con los profesionales, lo que refuerza la idea de que el embarazo se produjo sin planificación por falta de orientación y conocimientos específicos. Otros estudios ya han hecho referencia a la precariedad de la asistencia ofrecida a las mujeres con LES, destacando la falta de orientación sobre planificación reproductiva y el pesimismo profesional en relación con la combinación de LES y embarazo^(1,13)^. A veces, esta precariedad asistencial se debe al enfoque profesional en el tratamiento farmacológico de la enfermedad, lo que hace invisibles los deseos, los sueños y los ideales de estas mujeres.

Estas características asistenciales repercuten negativamente en la ocurrencia de embarazos no planificados y experiencias traumáticas^(1,2,3)^. Además, en presencia de SAF, el tratamiento se vuelve aún más complejo, ya que requiere la aplicación diaria de anticoagulantes inyectables, que provocan dolor, hematomas y sangrados^(20)^, lo que contribuye a una percepción negativa de la experiencia.

Así, la construcción del «yo» a partir de esta experiencia es, en su mayoría, negativa, ya que el proceso se vive de forma peculiar, impregnado de sentimientos de miedo, angustia e incertidumbre. La falta de interacción social o la interacción social deficiente con el equipo multidisciplinar produce impactos aún más negativos. Por lo tanto, es importante dar visibilidad a la mujer y a sus anhelos a lo largo del seguimiento, a fin de comprender que los deseos/sueños pueden cambiar dependiendo de la etapa en la que se encuentre la enfermedad y de los factores de riesgo involucrados.

Es importante destacar que la vigilancia de una mujer con LES debe realizarse desde el periodo preconcepcional hasta el puerperio^(5,21)^, con el objetivo de investigar de forma precoz los factores de riesgo y las enfermedades que pueden afectar a los resultados gestacionales^(5,13,22)^. El asesoramiento preconcepcional debe incluir orientaciones como: riesgo de exacerbación de la enfermedad y las posibles complicaciones obstétricas, fetales y perinatales asociadas al embarazo no planificado; riesgos de prematuridad; investigación de situaciones clínicas precoces; la importancia de la gestación con la enfermedad en remisión, así como la importancia del seguimiento prenatal de alto riesgo^(5)^.

Con el avance de la ciencia y el conocimiento científico sobre este fenómeno, la planificación reproductiva cobra cada vez más importancia y se recomienda, debiendo identificarse como una de las formas de reducir la morbimortalidad y aumentar el éxito de estos embarazos^(9)^. Así, la planificación de la gestación con LES y la vigilancia reproductiva de la mujer potencian las posibilidades de buenos resultados gestacionales, contribuyendo a la construcción de significados positivos.

Una posible limitación del estudio está relacionada con el sesgo de selección, ya que las mujeres con LES fueron localizadas en una plataforma virtual, lo que limitó la participación de aquellas que no tenían acceso a Internet. Sin embargo, los resultados de este estudio favorecen el conocimiento científico sobre la gestación en mujeres con LES y generan insumos para debates sobre la asistencia sanitaria dirigida a este público, así como para la comprensión de la importancia de la planificación reproductiva para las gestaciones con esta enfermedad crónica. Se sugiere que futuras investigaciones estudien el papel de la familia ante este fenómeno.

## CONSIDERACIONES FINALES

La construcción de esta teoría sustantiva concluyó que los significados atribuidos al embarazo por las mujeres con LES difieren en función de la experiencia vivida, que a su vez está influenciada por la planificación o no del embarazo. La experiencia de un embarazo planificado con apoyo multiprofesional en salud se considera positiva, ya que ofrece resultados gestacionales favorables. Por el contrario, el embarazo no planificado y vivido sin apoyo multiprofesional o con un apoyo frágil se considera negativo, especialmente por la experiencia de complicaciones, la construcción de sentimientos negativos y los resultados gestacionales desfavorables.

La interacción personal con el fenómeno se ve directamente afectada por la situación de la mujer con LES, haya planeado o no su embarazo, y por la interacción social establecida con el equipo multiprofesional de salud en su seguimiento en el proceso salud/enfermedad/embarazo. Estos factores influyen directamente en la forma en que la mujer percibe, se comporta y significa el embarazo. Por lo tanto, la planificación reproductiva se percibe como la clave del desenlace gestacional en el LES.

## Data Availability

El conjunto completo de datos que respalda los resultados de este estudio se publicó en el propio artículo.
